# Section on Surgery and Anatomy

**Published:** 1881-06

**Authors:** 


Adjourned.

### FIRST DAY.

SURGERY AND ANATOMY.—Chairman, Dr. Hunter McGuire, of Virginia; Secretary, Dr. Duncan Eve, of Tennessee.

Dr. Joseph H. Warren, of Boston, Mass., opened the work of the Section by exhibiting a collection of instruments devised by himself:

First. A few vermicular pointed catheters of three sizes. They combine the advantages of a screw and wedge, and its use is attended with less irritation and less difficulty of introduction. The next was his modification of Sir Henry Thompson's tube. It consists in having a tip, one-half of which is thrown back by a spring after it has entered the bladder. It acts as a scoop and allows of a free passage of the fragments. The tip may be made to revolve or be stationary. The straight tubes used by Bigelow may be made with this tip. It may be used to remove balls from gunshot wounds, foreign substances from nose, throat, or ear; for these cases smaller tubes may be used. A third instrument was a Bigelow tube with a vermicular point. Much larger tubes of this sort with less shock are passed. Many of the dangers of large instruments are overcome by its use. The writer names this the Thompson-American Tube, in honor of Sir Henry Thompson. The next shown was a uterine probe with revolving point. This has a fenestrated opening which can be threaded with a tuft of cotton. The next were a set of rapid dilating urethral and uterine sounds with revolving bulbular points, and also may be used for œsophagus and anus. The next was a trocar with revolving point—useful for tapping cases of general anasarca, etc. The next was a serrated herniotome ; hæmorrhage, with its use, is less. Dr. Goldenbird's torsion forceps were also exhibited. Also, a self-retaining trocar for paracentesis thoracis. The next was his latest devised hernia syringe, and finally an instrument called the demonstrator's knife—useful in dissecting veins, arteries and nerves. He also exhibited a modified sound which he thought possessed many advantages.

Dr. Lewis A. Sayre, of New York, did not think the sound possessed anything to recommend it.

Dr. Gouley, of New York, saw no advantage in the rotary spiral sound—not even simplicity to recommend it.

Doctor Burchard reported an interesting case of ulceration of the appendix vermiformis, with remarks upon abdominal section in cases of perforation of the bowel. The writer took grounds strongly in favor of operative interference, and based his opinions on experience in many cases. Dr. Burchard advocated early operative interference in inflammatory affections around the cæcum. The operation is easy. An exploratory incision can be made for diagnostic purposes. Referred to acute perforating typhlites. Thinks it should be divided into acute and chronic cases ; the acute require surgical interference. Also has noticed some cases which appear to be recurrent. Thinks it better to stitch the intestine to the abdominal incision and make an artificial anus ; and above all, thinks it our duty to give the patient the benefit of the operation with due antiseptic precautions.

Dr. James E. Reeves, of Wheeling, West Va., presented for Dr. B. W. Allen, of Wheeling, a report of a case of pyonephrosis, exhibiting a nephritic-calculus weighing 480 grains.

The case was that of a widow, age, 55 years. Her general appearance was that of a great sufferer. She was sallow, emaciated, asthmatic and constipated. Her bad health dated from her menopause. On examination a large tumor was found extending from the right hypocondrium to the left and to the pelvis. Fluctuation was marked. Aspiration evacuated 18 pounds of sero-purulent matter, and this was followed by immediate relief. It was now found that the tumor had no connection with the liver, and the history of the case forbade the idea of its being ovarian. The benefit from the operation was only temporary, and death resulted. An autopsy showed a tumor extending from the under surface of the liver with which it was in contact, but not adherent downward into the cavity of the pelvis. The lower end of the ileum ran across the tumor to the ileocæcal-valve, producing a constriction noticeable before death. The sac was fifteen inches in length, twelve inches wide, and six through. It contained ten pounds of purulent fluid, and was found to be the remains of the left kidney. An enormous renal calculus was found imbedded in the sac together with smaller ones, weighing 416 grains. At no time were there symptoms of uremic poison, and the other abdominal organs were healthy.

Upon motion, the section adjourned after the above report.

SECOND DAY.

SURGERY AND ANATOMY.—Chairman, Dr. Hunter McGuire, of Virginia ; Secretary, Dr. Duncan Eve, of Tennessee, met at Young Men's Christian Association Hall, at 3 o'clock, P. M.

Dr. Charles F. Stillman, of New York City, read a paper upon " A New System of Surgical Mechanics," illustrated by numerous drawings and instruments.

The system demonstrated by Dr. Stillman is based upon the principle of *local extension* as opposed to *general extension* developed by all other systems, which local extension is produced by the use of the sector splint in the various forms shown by him as adapted to the several joints. He first traced the history of this sector splint from the initial idea of two slotted and clamped strips, attached by copper plates, to its present improved and varied form. Having given a cursory mention of Buck's extension, various modifications of the long splints, Hutchinson's method and Thomas' plan, of Liverpool, he summed up the advantage of his system as follows:

1. Extension at any angle with motion.
2. Extension at any angle with luxation.
3. Fixation.
4. Motion complete or limited—constant or occasional.
5. Exposure of surface about the joint, admitting compression, elastic or otherwise, hot and cold applications, blisters, dressings and easy inspection.

This was followed by an exhibition of splints for the spine, hip joint, knee joint, ankle joint, and elbow. Also, an instrument for reducing cases of talipes in various forms and of long standing, by which instrument the surfaces of the tarsal bones are separated before the foot is made to assume a normal position.

Dr. Kinloch, of South Carolina, thought too much advantage was claimed for such contrivances; thought much could be done by rest secured in other ways; wished this fact was better appreciated; was sorry he could not share the enthusiasm of Dr. Stillman, but had failed to secure as good results.

Dr. Quimby, of Jersey City, N. J., endorsed fully Dr. Kinloch's remarks, and was especially emphatic in approving the treatment of club-foot with adhesive strips, plaster-of-paris, etc., instead of costly shoes and braces. Thought mechanical instruments were sometimes useful, but were also capable of great abuse, and thought that they did not control muscular contraction.

Dr. Alfred C. Post, of New York, read a paper on Plastic Operations on the Face. The first case which he related was one of epithelioma, involving the left side of the face, about 55 millimetres in diameter, extending above to a line within six millimetres of the margin of the lower lid, and below to a line midway between the ala of the nose and the angle of the mouth. The patient was a man 61 years of age. Dr. Post operated on the 15th of May, 1880, by making a horizontal incision, separating the morbid growth from the lower lid, and another parallel incision below the tumor, connecting the two by vertical incisions before and behind. He then dissected up from the adjacent parts the diseased mass, included between the four incisions, leaving a quadrilateral chasm, nearly of a square form. The two vertical incisions were then extended downwards, the anterior one to a little below the base of the lower jaw, and the posterior one nearly as low as the base of the jaw. From the inferior extremities of these two vertical incisions, curved incisions with their concavities looking backward and upward, were made to the extent of five centimetres, including between them a peduncle of skin and subjacent tissues for the nourishment of the extensive flap. The flap was then drawn up and secured by sutures so as to fill up the whole space left vacant by the removal of the disease. The wound healed throughout, leaving the patient but slightly disfigured. The second case was one of absence of the upper lip, and of the columna nasi, as well as of the portion of the right ala nasi, occasioned by the application of a caustic paste for the removal of a supposed cancer of the upper lip. The patient was a man 65 years of age.

Dr. Post operated on the 1st of May, 1880. He commenced by separating from the cheek a small remnant of the upper lip, which remained at the left extremity, and reversing it so as to form a columna for the nose. He then made two horizontal flaps from the cheeks with peduncles curved downward and forward, and united them by means of pins and sutures, so as to reconstruct the upper lip. A number of supplementary operations were required, and the result was a marked improvement in the appearance of the patient.

Water-colored drawings were exhibited, showing the conditions of these two patients before and after the operations.

They showed the benefit and added much to the interest of the paper.

Dr. D. H. Goodwillie, of New York city, read a paper on Treatment of Arthritis of the Temporo-Maxillary Articulation.

He reported cases treated. Causes of arthritis are local and constitutional. Whenever it occurs, the motion of the jaws becomes impaired according to the cause, severity, and length of the disease, and often requires long treatment to restore the muscles to their normal condition.

The treatment of the arthritis is done by means of an apparatus to relieve the joint of pressure on the inflamed articular surfaces.

It is made as follows: An impression of the teeth of either jaw is taken and an interdental splint made, the posterior part of which is raised a little for the purpose of a fulcrum, on which the back tooth of the opposite jaw rests.

Another impression is taken of the chin, and a rubber splint is made to fit it. A skull-cap is next made to fit the head closely, with elastic bands on each side passing down from it and fastened to the chin-splint.

The interdental splint is placed in position in the mouth, and the back teeth of the jaw closed on the fulcrum of the interdental splint; then, when pressure is made on the chin by tightening the elastic bands connected the skull-cap with the chin-splint, the joint is relieved from pressure.

Dr. Moore, of Rochester, called attention to cases where cicatricial bands caused the trouble—division of which usually cured them.

Dr. S. D. Gross, of Philadelphia, saw comparatively few such cases since the abuse of calomel had ceased; had not been able to accomplish much with wedges, etc.; alluded to section of the bone; had performed the operation.

Dr. Hunter McGuire, of Richmond, Va., said that one of the cases alluded to by Dr. Goodwillie afterward fell into his hands, and he was sorry to inform him that the case was not cured. There was no motion whatever. As every other plan had been tried by Drs. Sayre and Goodwillie, Dr. McGuire took out a wedge-shaped piece of bone just at the angle. The result was good. Also spoke of a case where a bridge of bone passed from upper to lower jaw for eleven years—no movements in joints; yet returned quickly after division of the bridge of bone. Thought with wedges, etc., the treatment should be prolonged.

Dr. Lewis A. Sayre, of New York, applied a cast upon a case of lateral curvature, and explained the action, etc.

Upon motion of Dr. S. D. Gross, a vote of thanks was tendered Dr. Sayre for his demonstration.

Dr. Hodgen, of St. Louis, objects to the jury mast, on the ground that the straps on the child retards the growth of the jaw. Thinks casts cause hernia.

Dr. Sayre said he had never seen any of the bad effects from the method.

Dr. Hutchinson was in favor of self-suspension, raisêd shoe on the opposite side, friction, massage, etc. Did not think much of this or any brace.

The Chair announced that he appointed Drs. Kinloch, of South Carolina; T. F. Pratt, of St. Louis, and Burgett, of New York, from the Surgical Section on the Prize Award Committee.

Dr. B. A. Watson, of Jersey City, N. J., read a paper, the title of which was "An Experimental and Clinical Inquiry into the Etiology and Distinctive Peculiarities of Traumatic Fever." The writer, in the first part of his paper, enters fully into the history of traumatic fever, presenting the views of Billroth, Wagner and Weber. He is of opinion that the numerous and ever-changing views of the etiology of traumatic fever, beginning with Hippocrates and coming down to our times, reflect for each age the true condition of the medical and allied sciences; but thinks it sufficient to classify all of the theories as the septic, nervous and nervo-septic. The question first to be answered is, What do we understand by traumatic fever? According to the writer's interpretation of the differentiations given by most writers, he thinks it unfortunate to use the term traumatic instead of septic, since many severe traumatisms are not attended with fever. Another objection is found in the fact that recent investigations in connection with the practice of antiseptic surgery suggest the probability of another form of fever developed in connection with wounds unlike that from septic absorption. The symptoms, such as stupor, unfitness for mental efforts, hallucinations, prostration, etc., are not noticeable in this form. Frequently the increased temperature is the only important clinical symptom. Patients with a temperature of 102°–104° go about with ease and amuse themselves. A patient with both arms amputated, treated by Lister's method, and whose wounds healed by first intention, walked all over the house on the day after the operation and continued to do the same with an axillary temperature of 104°. Another who had received a severe compound fracture of the leg, with extensive contusion and laceration of the soft parts from the knee to the malleoli, and who showed an axillary temperature of

105.8°, did not exhibit the slightest trace of illness. There can be no question of the propriety of this division into the septic and non-septic wounds. We observe putrefactive decomposition, septic absorption and septic fever in the wounds which are open to the air; but in those wounds which are purely subcutaneous, or where the antiseptic treatment has been successfully applied, we find only the non-putrefactive decomposition, harmless absorption and a non-septic fever, which has neither pathological nor prognostic significance. The term traumatic fever applied to the morbid condition due to septic absorption, is a misnomer, and it is questionable whether or not it should be applied to the mere septic form. An open wound is essential to the development of a septic fever; but its relation to the non-septic fever is not settled, and can only be studied in connection with antiseptic fever.

Regarding the influence of agents, such as chloroform, ether, and carbolic acid in lowering the temperature and influencing the production of fever, the writer had been experimenting with rabbits, of normal temperature, 102.7. After 30 minutes of complete anæsthesia with ether the temperature fell to 101.6°; in 30 minutes more it was to 101.9°; in one hour and a half it rose to 102.5°; in two hours to 102.2°. The average temperature of the rabbits to which chloroform was given was 103.2°; in thirty minutes it went down to 101.2°; one hour back up to 101.9°; and one hour and a half to 101.7°; in two hours to 101.2°; on the first day it was 102.3°; second day to 102.4°; third to 102.3°. The fall of temperature under ether was rapid but transient, passing off almost entirely in an hour; while the fall after the use of chloroform was marked and lasted three days; in this latter the fall is greater, and the reaction slower. In studying the influence of shock in traumatism, it was aimed to do so without organic lesions. One pole of a battery applied to the mouth and the other to the rectum of rabbits, whose normal temperature was 103.2°, showed, after eighteen hours of use, no reduction of temperature. In non-compound fracture of the rabbit's leg with a normal temperature of 103°; in second, third, and fourth hours, 103.5°; sixth, 103.4°; seventh, 103.6°. The highest average temperature occurred on the seventeenth day.

On the twenty-second day there was pretty firm union. Upon ten rabbits the experiments consisted in injecting into the lumbar region; every antiseptic precaution was taken. The average temperature before the injection was 103.6°; the average temperature three hours after, 103.6°; second day, 103.4°; third, 103.2°; fourth, 103.7°; fifth, 103.6°, and eighth, 103.6° The injections produced no rise of temperature above that produced by the simple fracture; in most cases absorption of the fluid; and swelling took place without a rise of temperature; in the exceptional case abscess with septic poison and death resulted. After these careful experiments, the writer is convinced that the fever is not due to the lesion, and raises the question as to whether or not it is due to the absorption of carbolic acid. The carefully-made experiments of Edelberg, inclines him to think that the traumal fever noticed in connection with the antiseptic treatment should be attributed rather to the partly congealed and partly fluid blood than to the carbolic acid. The writer, after reviewing the question thoroughly, is confident that traumatic fever is due to blood extravasation, but thinks it can only be due to carbolic action on the wound secretion aided by the air, while the absorption of the acid product is unquestionably the first step in the development of the morbid condition.

Upon motion, the Section adjourned.

THIRD DAY.

SURGERY AND ANATOMY.—Chairman, Dr. Hunter McGuire, of Virginia; Secretary, Dr. Duncan Eve, of Tennessee. Met at Young Men's Christian Association Hall, at 3 o'clock P. M.

Dr. B. A. Watson, of Jersey City, N. J., read his paper, an abstract of which appears above.

Dr. H. F. Campbell, of Georgia, asked if the writer recognized traumatic fever in wounds not antiseptically treated.

Dr. Watson replied that he had divided them into septic and non-septic.

Dr. Campbell appreciated the fact that traumatic fever may arise independent of septic absorption. He cited as an example the introduction of a catheter. If the patient is of a highly nervous temperament, and the day is inclement, he has often noticed a rise of temperature of an intermittent character, marked by a rise in the forenoon and a decline in the afternoon; this fever also is recurrent. He called this traumatic; others defined it urethral. He saw in such cases an intimate relation between the wound and brain through nerves of the wounded part, and must think this reflex action through the spine, in some way a factor of this fever. He has not extensively used antiseptics, but wished to call attention to his treatment of patients after operation. In his section—and he thought it applied to most malarial sections—he has noticed that quinine, after an operation, almost invariably cut short the fever. He was so much impressed with its value that he never neglects to use it after operations. Regarding its use before an operation to prevent shock, etc., as practiced by Dr. Hunter McGuire, he knew but little, but felt warranted in advising its use after an operation.

Dr. Nancrede, of Philadelphia, spoke of the use of antiseptics.

Dr. Alfred Post, of New York, called attention to the plan of Professor Markoe, and thought it superior to that practiced by Lister. After trying both plans, he preferred Markoe's.

Dr. Quimby, of Jersey City, also spoke in favor of Markoe's plan. He had seen Listerism practiced by Lister in his own hospitals, without seeing better results than Markoe's afforded.

Dr. Charles A. Leale, of New York, read an original paper on labial carbuncle or malignant pustule of the lip, giving the etiology of the malady, and showing from the writings of Billroth, Paget, Gross, Parker, and others, the extremely fatal nature of the disease, and with what solicitude they treated a patient so afflicted. Dr. Leale described the anatomical relation of the blood-vessels, nerves, lymphatics, and structures, in which carbuncle usually appears, and the *modus operandi* by which the septic or purulent matter affects the contiguous lymphatics and veins, causing metastatic adenitis, phlebites, and frequently gangrene, ending in death by asthenia or heart-clot.

Dr. C. A. Leale's method of treatment of labial carbuncle, is to examine thoroughly the extent of the subcutaneous induration by the tactus eruditus, bearing in mind the location of the arteria septi nasi, as it passes down near the border of the lip to join the superior coronary artery avoiding each, make a free incision outwards and downwards along the course of the fibers of the obicularis oris muscle extending the cut each way in a line until all the diseased tissue has been passed, taking care not to go through the mucous membrane lining the lip, to which extent the disease rarely extends. Dr. Leale then, with a fine piece of ivory or wood, with its ends covered with cotton, thoroughly applies to all the diseased parts and cut surface, the chemically pure nitric acid; this is pressed with sufficient force into all the diseased parts, so that every little pocket of pus is reached, and the intervening membranes destroyed, which would otherwise be left to slough off and continue the septic or purulent infection.

Dr. Leale stated that by this time the nervous and constitutional systems are very much depressed, and for the relief of the fever pain, he gives morphia *pro re nata*, and to overcome the blood poisoning, whisky is given liberally, largely diluted with water.

Dr. Leale stated that on some occasions he has had to apply the acid on the second or third day, or until all the poison has been rendered inert. His subsequent treatment is by the open wound method, applying unguentum balsam Peru, lightly smeared on lint, and giving the patient the most nutritious diet and restorative tonics.

Dr. Leale claims that by this treatment all the little canals making the cut surface appear like a sieve are reached, and that the entire poisonous mass is kept within circumscribed boundaries, and the absorbed poisons by sustaining the system are eliminated. In the early part of the treatment, a full dose of sulphate of magnesia, largely diluted in water, is given. As a rule, it will be found that on the third or fourth days after the incision and the first application of the acid, all danger will have subsided and the convalescence will steadily progress.

But, in some instances, we may have acute mania from cerebral meningitis or erysipelas; the former to be treated by large hypodermic injections of morphia, and the latter, when possible, to be relieved by cooling lotions of lead and opium.

*Recapitulation.*—Carbuncle of the lip or malignant pustule of the lip has, by the usually recommended modes of treatment, proved fatal in a very large proportion of cases, even in healthy, strong and vigorous adults, and is, therefore, a much dreaded malady. The cause of the failure, in Dr. Leale's opinion, is that, by the usual method of treatment, only a portion of the diseased mass is reached, and consequently the remaining imprisoned *materies morbi* cannot be eliminated, nor can any local application reach the disease.

Dr. Alfred C. Post, of New York, stated that the subject was a most important one, and was very much pleased with the paper of Dr. Leale. Dr. Post thought that when we see patients early with malignant pustule a large proportion can be saved. He usually operated by cutting through the vermillion border of the lip.

*Resolved*, That the President of the Surgical Section appoint a committee of three, to select six subjects for presentation and discussion, at the meeting of the Section in 1882, assigning each subject to a gentleman, who shall, by the reading of a paper, or otherwise, introduce his subject for discussion.

*Correction.*—Dr. Hutchison *advocated*, and did not, as was stated in yesterday's report, oppose the use of Dr. Sayre's method of treating spinal curvatures. He only mentioned its liability to cause hernia. He uses the cast frequently.

